# A Data-Driven, Mathematical Model of Mammalian Cell Cycle Regulation

**DOI:** 10.1371/journal.pone.0097130

**Published:** 2014-05-13

**Authors:** Michael C. Weis, Jayant Avva, James W. Jacobberger, Sree N. Sreenath

**Affiliations:** 1 Department of Electrical Engineering and Computer Science, Case Western Reserve University, Cleveland, Ohio, United States of America; 2 Case Comprehensive Cancer Center, Case Western Reserve University, Cleveland, Ohio, United States of America; Florida State University, United States of America

## Abstract

Few of >150 published cell cycle modeling efforts use significant levels of data for tuning and validation. This reflects the difficultly to generate correlated quantitative data, and it points out a critical uncertainty in modeling efforts. To develop a data-driven model of cell cycle regulation, we used contiguous, dynamic measurements over two time scales (minutes and hours) calculated from static multiparametric cytometry data. The approach provided expression profiles of cyclin A2, cyclin B1, and phospho-S10-histone H3. The model was built by integrating and modifying two previously published models such that the model outputs for cyclins A and B fit cyclin expression measurements and the activation of B cyclin/Cdk1 coincided with phosphorylation of histone H3. The model depends on Cdh1-regulated cyclin degradation during G1, regulation of B cyclin/Cdk1 activity by cyclin A/Cdk via Wee1, and transcriptional control of the mitotic cyclins that reflects some of the current literature. We introduced autocatalytic transcription of E2F, E2F regulated transcription of cyclin B, Cdc20/Cdh1 mediated E2F degradation, enhanced transcription of mitotic cyclins during late S/early G2 phase, and the sustained synthesis of cyclin B during mitosis. These features produced a model with good correlation between state variable output and real measurements. Since the method of data generation is extensible, this model can be continually modified based on new correlated, quantitative data.

## Introduction

Cell cycle research expanded dramatically after the discovery of cell cycle regulating genes in yeast in the 1970’s [1]. A review of yeast studies identified 475 proteins participating in 732 reactions [2]. The mammalian cell cycle is more complicated, and as more biochemical reactions are discovered and incorporated into a narrative model, the ability to intuitively predict outcomes after perturbation should diminish. Mathematical modeling forces formalization of concepts in a precise language, and models allow analytic evaluation of complex system behavior. Eventually, as a model becomes more accurate, hypotheses can be tested in silico. That is the promise of “Systems Biology” [3].

The cell cycle is a regulated, ordered sequence of compartmentalized, simultaneous (parallel) and serial chemical reactions [4]. Presumably, order and compartmentalization are underlying features, necessary for genomic stability, balanced cell growth and division, and tissue formation. At one level, the cell cycle control system is manifested physically by a large set of interacting biomolecules. In particular, the “backbone” of the system is the sequential activation of a series of cyclin-dependent kinases (Cdks). Cdks were among the first discovered proteins/genes that evoked the logic of the control system.

Cdks are regulated by three mechanisms: 1) cyclin availability - the kinase subunits are expressed at high levels throughout the cell cycle, but activities are regulated by oscillating levels of activating cyclin partners; 2) phosphorylation – all cell cycle Cdks are activated by Cdk activating kinases (CAK), and some cell cycle Cdks are inhibited by Wee1 and Myt1 kinases or promoted by the Cdc25 phosphatases, and 3) peptide inhibitors - active cyclin-Cdk complexes are inactivated by binding Cdk inhibitors (CKIs) such as the INK4 (p16, p15, p18, p19) and CIP/KIP (p21, p27, p57) gene families [5], [6].

Cyclin concentrations are determined by the opposing rates of synthesis and degradation. Synthesis depends on specific transcription factors and degradation depends on ubiquitin-dependent proteolysis systems. Both are regulated by additional controls. Coupled transcription/translation and degradation sequentially orders the periods of high concentration of specific cyclins. The levels of CKIs also depend on their production rate, which is governed by regulated transcription and destruction rates, which in part depend on the activity of Cdks (Cdk-phosphorylated CKIs are rapidly ubiquitinated and degraded). The interplay between these systems shifts the cell sequentially through genome and centrosome duplication, and chromosome and centrosome segregation.

We have identified >154 mathematical models of the cell cycle (a large subset of these are listed in [7], also see Tables S1 and S2 in File S1). Most models have focused on understanding small, often hypothetical, parts of the cycle [8]–[12]. Others have focused on specific cell cycle phases and transitions, such as the G1/S transition and the G1 restriction point [13]–[20], or “G2+M” transition [21]–[24]. Recent models [17], [25], [26] have begun the difficult task of assembling larger scope models aimed at a regulatory system governing complete cell cycle transit. Coincident with the use of yeast as the primary model organism in cell cycle research, models of yeast cell cycles dominate the field [27]–[36]. Other models use the language and information from frog eggs [22], sea urchins [37], drosophila [38], and mammalian cultured cells [14], [17], [25], [39]. Some efforts have been aimed at a generic eukaryotic cell description [26]. Published cell cycle models cover a wide range of purposes and techniques. The large majority use ordinary differential equations (ODEs) based on chemical mass action assumptions. However, stochastic [40], time delay [41], partial differential equation models of spatiotemporal dynamics [42], Boolean logic [43], and hybrid models [44] have also been reported.

Most models are unsupported by quantitative biological data. While these models force a formal description and test the validity of hypotheses based on ideas from biology, especially on feedback mechanisms, hysteresis, and bifurcation in cell cycle control, there have been few attempts to fit these models to quantitative measurements [45]. In tandem with common biological reasoning, most models have been calibrated or validated against “qualitative” observations or general information about the timings of events. Model parameters are loosely tuned to produce stable sequences of oscillations in molecules known to be active in corresponding cell cycle phases. The use of genetic perturbations for qualitative validation of the proposed system structure extends this approach. Chen et al. [27], for example, utilized over 100 genetically engineered strains to confirm the canonical “wiring diagram” of the yeast cell cycle and constrain the calibration of a computational model. The use of quantitative dynamic data in models of the cell cycle is rare. Exceptions include the use of Xenopus laevis embryo extracts [11], [46], limited measurements of yeast proteins [47], [48], and synchronization, timed sampling, and immunoblotting of human cell lines [14], [39]. One effort used flow cytometric data, similar to that used here, but in conjunction with a hybrid model [44]. Here, we evaluated two mammalian models with correlated cytometric measurements of cyclin A2, cyclin B1, and phosphorylation of histone H3 at serine 10 (PHH3). However, to achieve agreement between model and data, we combined the two and structurally modified the new model.

**Figure 1:**
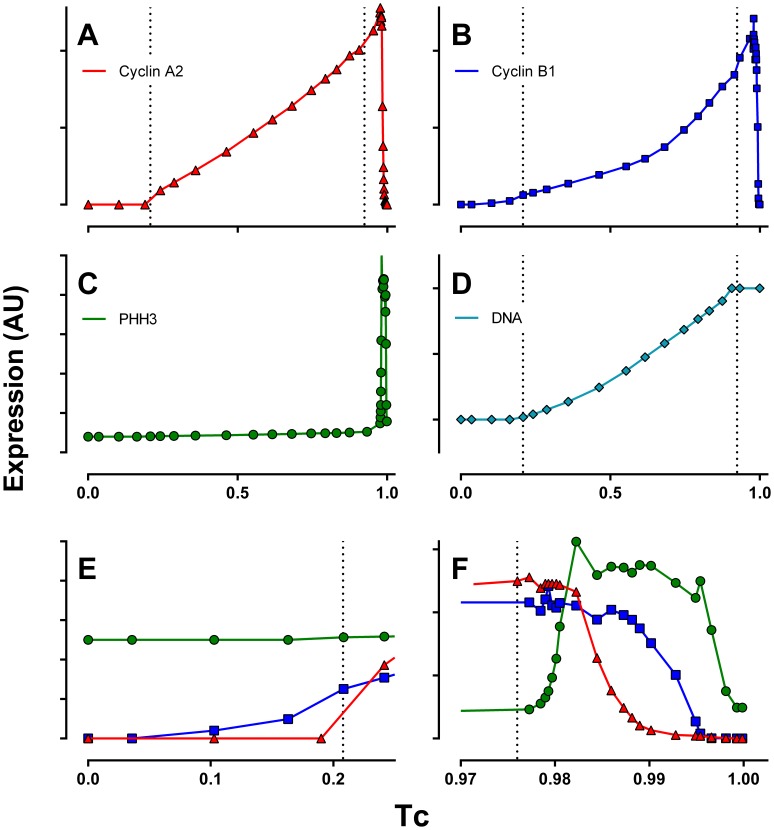
Cell cycle expression. Normalized expression profiles of cyclin A2 (**A**), cyclin B1 (**B**), and PHH3 (**C**), and DNA content (**D**). A magnified view of the first 25% of the cell cycle is presented in **E**, and the final 3% of the cell cycle in **F**. K562 cells were stained, measured by flow cytometry, and sample data were analyzed to produce expression profiles as described in Materials and Methods. Dotted lines demark the G1/S, S/G2, and G2/M boundaries as determined by cell cycle analysis of DNA content and gated enumeration of PHH3 positive mitotic cells.

It has been difficult to obtain precise quantitative data of cell cycle regulatory proteins. Cultured cells are naturally asynchronous and so most approaches use synchronization and relative, visual estimates of quantities or densitometry to quantify immunoblots to measure oscillating proteins in timed sampling over the course of one or two cell cycles. For mammalian cells, synchronization by physical or chemical means is always less than perfect and synchronization of many cell lines by chemical means results in altered cyclin expression [49], growth imbalance [50]–[52], and altered transit times [53]. The resulting data provide a good sense of expression versus time, but are imprecise both with respect to relative “normal” quantities and precise timing of cell cycle related oscillations for epitopes that will later be state variables in mathematical models. However, using quantitative cell-based methods, the information for the programmed expression of these same state variables is contained in a single, randomly sampled, asynchronous population. Asynchronous cells are distributed at each state within the data space of the programmed expression of said state variable. Therefore, because the cell cycle is a closed loop, single cell measurement data contain a sampled version of the dynamic expression profile of the measured molecules [54]–[57]. In a cycling population, the frequency of cells in each phase or state is proportional to the time spent in that state. In cell cycle flow cytometry, if correlated parameters are chosen to unambiguously isolate a continuum along an expression profile, that expression profile can be unambiguously derived. Guided by heuristics assembled from canonical cell-cycle knowledge, we have derived a methodology to extract the embedded dynamic profiles of cell-cycle proteins from statically sampled, multiparameter cytometry data. This approach is illustrated in previous publications [54]–[57] and the results for K562 cells are presented in Figure 1. With this method, time is relative and expressed as a fraction of cell cycle time (Tc). To convert to real time values, measurements of population doubling time can be used to estimate Tc and the time scale transformed accordingly. The doubling time of K562 cells has been reported as 24–30 hours [58]; under our growth conditions the rate is variable - we have measured it as 18 and 22 hours. A reasonable guess for Tc in Figure 1 might be at the higher end of values (e.g., 30 hours), since G1 accounted for only 21% of Tc, which would equal a T_G1_ of 6 hours.

In the work presented herein, our goal was to ask whether calibrating an ODE-based cell cycle model to correlated, dense expression data forced a substantive modification. We focused on the mitotic cyclins because the expression of cyclin A2 and B1 are, in general, very similar in immortalized, transformed, tumor-derived cell lines, and primary cells (e.g., stimulated lymphocytes) and also similar between differentiated cells (epithelial, mesenchymal, hematopoietic, etc.). We found that while published models capture the major features of mitotic cyclin expression, modifications were required to achieve agreement between model dynamics and quantitative expression data.

## Results

Figure 1 presents the expression profiles of cyclin A2, cyclin B1, and PHH3 in the K562 cell line. Cyclin A2 synthesis begins at or near the start of S phase (Figure 1E; for further corroboration, see the supplementary data and discussion in [44]). Synthesis continues into M, wherein it abruptly decreases as expected (Figure 1F). The data show a two-phase increase – approximately linear through S phase, followed by a rapid increase in expression in G2 phase (Figure 1A). The pattern of cyclin B1 expression is similar, however, its expression begins earlier than cyclin A2 (Figure 1E), and the primary increase in expression is non-linear and the secondary rate of increase is more rapid than cyclin A2 (Figure 1B). Cyclin B1 decays later in mitosis than cyclin A2 (Figure 1F). PHH3 is a marker of mitotic cells, increasing very early and remaining elevated through to cytokinesis. Histone H3 is likely phosphorylated at serine 10 by Aurora kinase B during mitosis [59]–[61], and any link between Cdk1 and Aurora kinase B activations is indirect. Since cyclin B1/Cdk1 is also activated at the beginning of mitosis, the onset of cyclin B1/Cdk1 activity should be approximately coincident with the onset of elevated PHH3 expression during early mitosis. While we did not measure cyclin B1/Cdk1 activity directly, we used PHH3 as a proxy for the timing of cyclin B1/Cdk1 activity.

Having obtained these expression profiles, our goal was to determine how closely published models and rate constants matched the data, and if the fits were not close, what changes were needed in existing models to make them fit – in essence, we were attempting to calibrate the published models.

**Figure 2:**
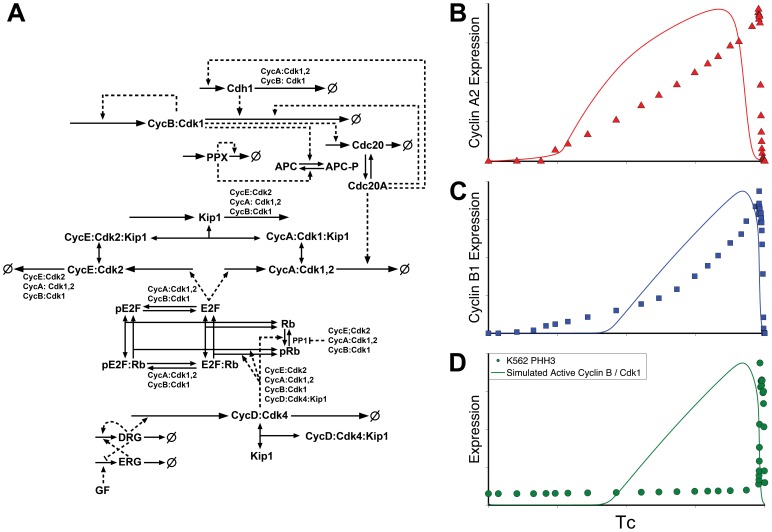
Model Schematic and comparison of model outputs to expression data I. **A**: schematic derived from the Conradie model [25]. Solid lines indicate chemical reactions and dashed lines indicate reaction modification (regulation). **B, C, D**: normalized comparison of model outputs with K562 data. Simulations were carried out using the published protocols and kinetic rate constants. Since antibodies do not discriminate between active/inactive, free/bound levels, total protein amounts (bound and free, inactive and active) from the model were compared to K562 measurements. As discussed in the text, PHH3 is used as a proxy for the timing of cyclin B/Cdk1 activity onset (identical to total cyclin B/Cdk1 expression in this model).

**Figure 3:**
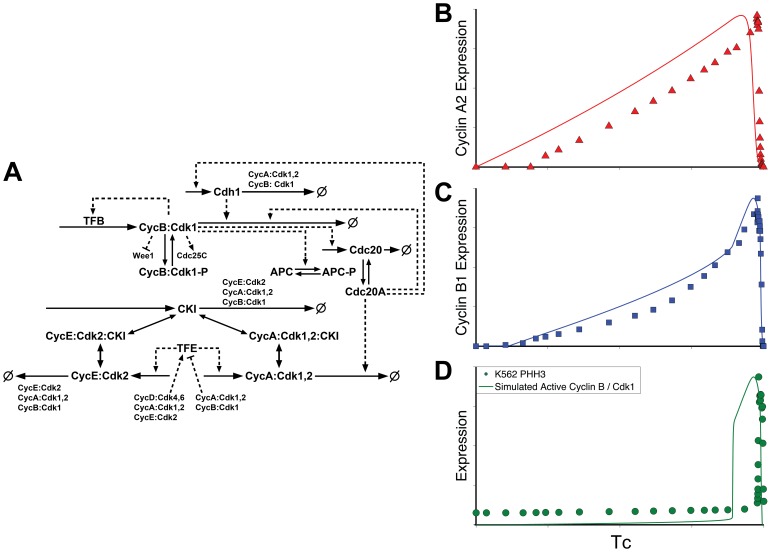
Model Schematic and comparison of model outputs to expression data II. **A**: Schematic derived from the Csikasz-Nagy model [26]. Solid and dashed line representations are as in Figure 2. **B, C, D**: normalized comparison of model outputs (simulated total protein levels (as in Figure 2) of cyclin A and cyclin B, and cyclin B/Cdk1 activity) to K562 expression data. Simulations were carried out using the published protocols and kinetic rate constants.

We tested two existing models, both of which captured canonical knowledge. They incorporated growth factor-induced activation of D cyclins that phosphorylate Rb, activating E2F, and resulting in the synthesis of E, A, and B cyclins. E cyclin initiated the deactivation of APC/Cdh1 and allowed cyclins A and B to accumulate. At the end of the cycle, B cyclin activated APC/Cdc20, which in turn degraded cyclins A and B, completing mitosis and resetting the cell cycle control system. Both models also incorporated the antagonistic relationship between a CKI, such as p27, and the cyclin/Cdks. However, beyond these basics, there were key differences between the models and each captured different aspects of the K562 expression profiles for cyclins A2 and B1, as shown in Figures 2 and 3.

The most recent comprehensive model of the mammalian cell cycle, by Conradie et al. [25], appears to be an updated version of that used to investigate restriction point control [17]. It therefore incorporated detailed mechanisms and equations for cyclin D activation, E2F interactions with Rb, and the role of cyclin E. Notably absent from the model were Wee1 and Cdc25, which regulate cyclin B/Cdk1 activity, or any mechanisms to account for the net rates of synthesis for cyclins A and B during interphase. Figure 2 presents a diagram of this model’s interactions and a comparison of the published model outputs with K562 derived expression profiles. The model captured basic trends in the expression of cyclins A and B, but the model outputs did not display the two distinct biphasic rates of synthesis evident in the K562 data. Furthermore, because the model lacked Wee1/Myt1 and Cdc25, it did not capture the delayed activation of Cyclin B and abrupt onset of mitosis demonstrated by the PHH3 expression profile.

The second model we examined is the mammalian implementation of a “generic cell cycle” model by Csikasz-Nagy et al. [26]. This model did not include any entry point signaling, and included limited dynamics of cyclin D (modeled as an initial concentration which grows exponentially) and E2F activation (approximated with an ultrasensitive Goldbeter-Koshland function [62]). The model included more comprehensive dynamics for the core downstream modules including cyclins A and B and incorporated Wee1 and Cdc25 regulation of cyclin B/Cdk1. Additionally, a G2 transcription factor was modeled for cyclin B, to implement secondary, auto-catalytic transcriptional control. These interactions and a comparison of the published model outputs to our data are shown in Figure 3. Cyclin B and activated cyclin B dynamics were captured fairly well with the model. Cyclin A dynamics started immediately and were only transcribed at one characteristic rate throughout the cycle. The K562 data, however, demonstrated a delay in cyclin A expression for the first 21% of the total cell cycle time and showed an increase in expression at the start of G2.

Although the published model state variable outputs do not fit our expression profiles well, both models captured canonical cell cycle dynamics. To better fit these models to our data, we used the ideas of Chen et al. [27] and worked backwards through the hierarchy of model assumptions: numerical rate constants, mathematical approximations, and the underlying biological network. However, without structural changes, we were unable to satisfactorily calibrate either of these models with our data. The outputs in Figures 2 and 3 represent the published models and parameters.

Manual and automated parameter optimization routines failed to significantly improve the fit between the previous models and our data [25], [26]. However, while a framework exists for applying mathematical optimization to the estimation of parameters via the so-called inverse problem, biochemical models are usually non-convex and multi-modal [63]. Additionally, functional relationships often exist between parameters such that each cannot be uniquely determined from a given set of observations [64]. Despite its critical importance, few methodologies exist for examining these dependencies *a priori* [65]. Calibration therefore requires broad and repeated searches through the parameter space [14], [66], [67]. State of the art algorithms often couple global stochastic searches (to cover space and leap local minima) with deterministic local methods (to refine the broad search). We tried three global algorithms: simulated annealing [68], a genetic algorithm [69], and the stochastic ranking evolutionary strategy (SRES) [70]. We searched both the entire parameter space and subspaces defined by the most sensitive 10% and 30% of the parameters. Despite evaluating well over 10^5^ parameter sets with each of these methods, we were unable to obtain satisfactory fits. This is most easily explained by the model structures, which define the space of possible system trajectories and preclude capturing certain data features (for example, the increase in cyclin A2 expression during G2). It bears noting however, that it is impossible to fully invalidate a model structure through calibration attempts alone. Algorithms are imperfect (in this case convergence is purely stochastic) and there are nearly infinite combinations of parameters. Nevertheless, our results strongly implied that certain unmodeled dynamics were likely significant in capturing features of the data.

**Figure 4:**
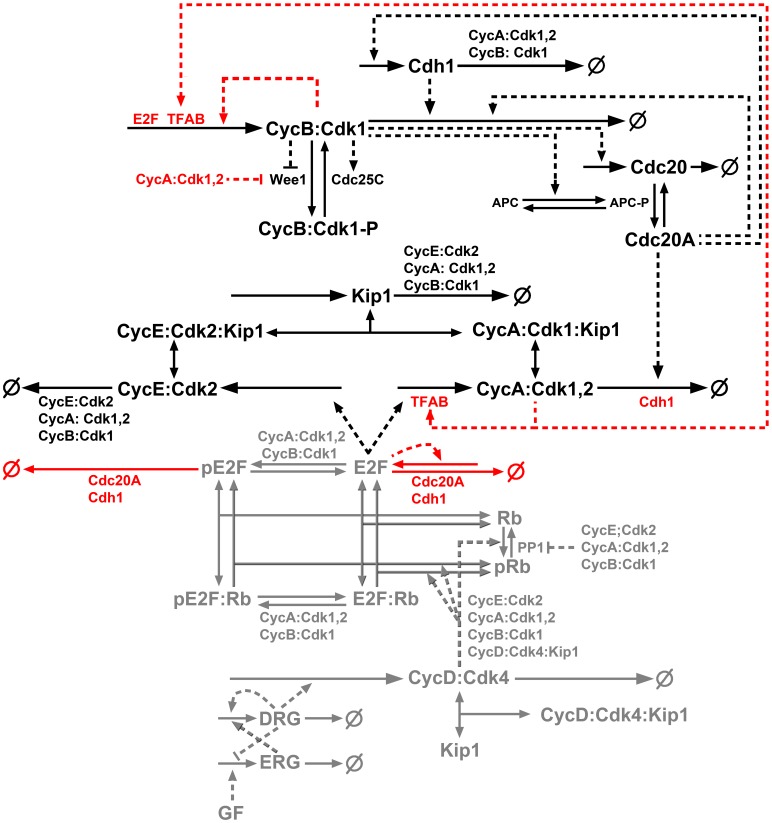
Diagram of a new model. The models presented schematically in Figures 2 and 3 were combined and modifications were introduced to provide better fits to the K562 data. The schematics of the original Csikasz-Nagy model [26] is shown in black, the Conradie model portion [25] is shown in gray, and the new modifications from this study are presented in red. Solid lines indicate chemical reactions and dashed lines regulatory effects. The apparent autocatalytic regulation of cyclin B/Cdk1 synthesis here represents a stabilization of B cyclin synthesis dependent on active cyclin B/Cdk1, whereas the same representation in Figure 2 (Conradie model) represents a Hill equation that is dependent on cyclin B, which is removed here. Thus, the dashed arrow represents a new modification here.

We obtained a better fit to data by modifying a published model. The Csikasz-Nagy model [26] provided the closest fit, and we therefore used this model as a base for this study and modification. The simulated output of cyclin B was similar to our data, but simulated cyclin A synthesis was unsatisfactory. In the next sections, we discuss modifications that were made to improve the model fit. The modified model was calibrated manually, using the methods and software listed in Materials and Methods, the pathway diagram in Figure 4, and differential equations provided in File S2 (Table S3) as guidance to adjust the strengths of relevant rate equations.

For most, perhaps all, of G1, we did not detect cyclin A2 expression in K562 cells. As shown in Figure 3 however, cyclin A in the Csikasz-Nagy model was produced throughout G1. The measured delay in cyclin A2 is due to at least two mechanisms. Firstly, transcription is delayed by the requirement for cyclin D to inhibit Rb and free the E2F transcription factors. Secondly, cyclin A is actively degraded by the APC/Cdh1 complex [71].

The Csikasz-Nagy model did not include detailed mechanisms of cyclin D, Rb, or E2F, but instead modeled the activation of E2F using a Goldbeter-Koshland approximation. E2F therefore switched on abruptly near the start of the simulation and remained at a constant level until switching off abruptly near the end. While lacking in its ability to capture cyclin A and cyclin B dynamics, an advantage of the Conradie model is its detailed dynamics of E2F, Rb, and cyclin D. The model decomposed the Goldbeter-Koshland function, used in an earlier incarnation of the model [17], into elementary mass action reaction rates and represented E2F activation in greater detail. Furthermore, cyclin D was modeled in more detail, incorporating transcription and proteolysis, as well as binding to a CKI such as p27. To better model G1 phase and the restriction point, we therefore replaced the Csikasz-Nagy mechanisms with those from the Conradie model. As an added benefit, the model incorporated “highly stylized” dynamics for basic upstream signaling pathway activity (representing, for example, MAPK), which provides an entry point for future, more complex models that include growth factor signaling, an essential element of cell cycle regulation.

**Figure 5:**
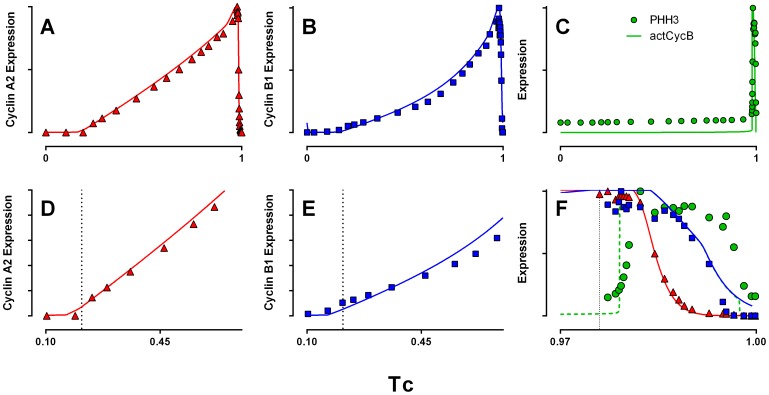
Normalized comparison of the modified model outputs to K562 data. **A, B, D, E, F**: total simulated levels (bound and free, inactive and active) of cyclins A and B, and cyclin B/Cdk1 activity (**C**) are compared to the measured expression profiles of cyclins A2 and B1, and PHH3. **D, E**: magnified view of early cyclin A and B expression. **F**: magnified view of the final 3% of the cell cycle. Symbols = data; lines = simulated output. The correlation between PHH3 expression and B cyclin/Cdk1 activity is for mitotic onset only. Since PHH3 is a proxy for B cyclin/Cdk1 activity, there isn’t a rationale for an exact match to the shape on the front side (onset) and we expect the activity to decrease as cyclin B is degraded, whereas it is known that phosphorylation levels of histone H3 (PHH3) decrease after B cyclin degradation (e.g., [59]).

Also, the Csikasz-Nagy model did not include degradation of cyclin A by APC/Cdh1. Other mechanisms such as Skp2 likely contribute to the proteolysis of cyclin A during G1 [72], but including Cdh1 regulation provides an effective and easily incorporated modification. As shown in Figure 5, including this regulation allowed us to model the delay in detectable cyclin A2 levels until close to the start of S phase. Biologically, this second mechanism is likely dominant, since cells like DU-145 with inactivating mutations in Rb (resulting in constitutively active E2F) do not express appreciable cyclin A2 in G1 [73].

As evident in Figure 1B, the synthesis of cyclin B1 during S phase is markedly nonlinear. The Csikasz-Nagy model considered two sources of cyclin B synthesis: constitutive, which synthesized cyclin B at a constant rate, independent of cell cycle progression; and enhanced, autocatalytic transcription during G2. Neither of these rates are capable of producing the S phase nonlinearity evident in the K562 data. However, there are a large number of transcription factors that activate cyclin B synthesis. E2F is among these [74] and represents an easily incorporated modification. E2F was modeled, in the Conradie portion adopted here, as active at nearly constant levels throughout S phase, without independent synthesis or degradation terms (only converted between active and inactive forms at the beginning and ends of the cell cycle). Therefore, simply tying cyclin B synthesis to E2F does not significantly change the character of cyclin B expression. However, E2F is autoregulatory and E2F promoters contain E2F binding sites [75]. This provides a mechanism where E2F may increase nonlinearly throughout the cell cycle. Data are not available to precisely tune the shape of E2F expression, but assuming autocatalytic synthesis of E2F and E2F-regulated synthesis of cyclin B allowed the model to be calibrated much more closely to the K562 data.

E2F regulation is quite complex, consisting of both activator (E2F1-3) and repressor (E2F3b,4-8) isoforms. Besides regulation through Rb binding, A and B cyclin/Cdk complexes phosphorylate E2F1-3. Phosphorylation by cyclin A/Cdk2 has been shown to inhibit the DNA binding of E2F1 and E2F3 (and mostly likely E2F2) [76], [77], while cyclin B/Cdk1 phosphorylation does not seem to have an appreciable effect [76]. However, APC/Cdc20 and APC/Cdh1 have been shown to target phosphorylated E2F1 for degradation during mitosis [78]. Both A and B cyclin/Cdks may prime E2F for this step, via phosphorylation and release from its transcriptional partner DP.

The E2F portion of our model, adopted from [25], captured these steps in broad strokes, including the phosphorylation of E2F by cyclins A and B. However, the model did not consider the synthesis or degradation of E2F. We added the autocatalytic synthesis of E2F to reproduce the character of cyclin B1 synthesis. In addition, we’ve incorporated the degradation of phosphorylated E2F by Cdc20 and Cdh1. This step was necessary to create a stable model that would repetitively cycle. To maintain the increase in expression needed to fit our measured increase in cyclin B1, the model required minor cyclin A/Cdk regulation of E2F compared to cyclin B/Cdk1. A more accurate representation of E2F dynamics requires some validating data. In particular, measurement of E2F levels, phospho-E2F levels, and E2F activity as a function of the cell cycle are needed to validate and refine the E2F dynamics modeled here.

The Csikasz-Nagy model considered cyclin B/Cdk1 as the key antagonist of Wee1 and activator of Cdc25, and created an ultrasensitive, bistable, feedback cycle where cyclin B/Cdk1 essentially activated itself. Ultrasensitivity has been identified in several biochemical systems [79], [80] and competition between Wee1 and alternative cyclin B/Cdk1 substrates generates ultrasensitivity in Xenopus oocytes [81]. However, in mammalian cells, evidence has been accumulating that Wee1 inactivation may be triggered by cyclin A/Cdk [82]–[84]. It has similarly been suggested that ultrasensitivity may arise from the competition between Wee1 and other cyclin A/Cdk substrates in human somatic cells [84]. The Csikasz-Nagy model approximated Wee1 activity with a Goldbeter-Koshland function, and so ultrasensitivity was inherent. Here, we modified the driving force of the inactivation from cyclin B/Cdk1 to cyclin A/Cdk to update the model in accordance with recent literature. Once Wee1 is inactivated, cyclin B/Cdk1 activates Cdc25 in an autocatalytic loop, which is responsible for the rapid activation of cyclin B/Cdk1. The inactivation of Wee1 is a trigger mechanism that allows a small amount of active cyclin B/Cdk1 to accumulate and start an abrupt autocatalytic rise in activity.

Cyclin B1 has constitutive and cell-cycle dependent transcriptional start sites [85]. As a key regulator of mitosis, cyclin B1 transcription is known to continue through G2. Key transcription factors include USF, NF-Y, B-Myb, and FoxM1 [86], [87]. In addition to constitutive synthesis, a mechanism for elevated G2 transcription of cyclin B was already in the Csikasz-Nagy model. This secondary transcription was modeled as being dependent on active cyclin B/Cdk1 levels. While it is known that cyclin B/Cdk1 can indirectly influence its own transcription, for example, through the activation of Bora, Plk1, and FoxM1, it is difficult to see how it could accomplish significant effect prior to appreciable cyclin B/Cdk1 activity, without invoking non-catalytic activity. Further, FoxM1 has been shown to require the phosphorylation of an autoinhibitory domain by cyclin A/Cdk before the transcription of cyclin B and other G2 phase targets [88]. Similarly, B-Myb also requires activation by cyclin A/Cdk [74], [89], [90]. This evidence supports basic reasoning that cyclin A/Cdk, which is already active, might drive the accumulation and activation of cyclin B. We therefore modeled the G2 cyclin B transcription factor (TFAB) as dependent on cyclin A/Cdk both to keep the model consistent with current literature, and to serve as a testable hypothesis for future cell cycle studies.

Similar to cyclin B1, our data indicate a change in the net synthesis rate of cyclin A2 in late S/G2. We therefore also propose, and modeled, two phases for cyclin A transcription. Without evidence, we assume cyclins B1 and A2 share a common transcription factor - although this transcription factor has a lesser effect on cyclin A2 compared to cyclin B1. This is not unreasonable - E2F, NF-Y and B-Myb, for example, have been shown to bind both cyclin A and cyclin B promoters [74], [90].

The modifications thus far discussed improved the simulation of interphase relative to the K562 expression profiles. Turning to mitosis, we encountered an additional inconsistency. The Csikasz-Nagy model degraded cyclins A and B simultaneously upon the activation of the APC/Cdc20 complex. To our knowledge, this is the case for all published differential equation models that include mitotic cyclin degradation. However, Figure 1 clearly illustrates the known delayed degradation of cyclin B1 relative to cyclin A2. There are several possibilities which might explain this.

It has been shown that cyclin B is subject to the spindle assembly checkpoint (SAC), while cyclin A is not. Cyclin A is degraded soon after APC/C phosphorylation by active cyclin B/Cdk1, while cyclin B and other critical mitotic proteins (e.g., securin) are protected by SAC-promoted sequestering of Cdc20 by Mad2 and the formation (with BubR1 and Bub3) of the inhibitory mitotic checkpoint complex (MCC) which binds to and inactivates the APC/C [91]. Cyclin A is degraded regardless of SAC activity by either or both of two proposed mechanisms: cyclin A may have a high enough affinity to compete with Mad2 for free Cdc20 before being targeted to the APC/C by Cks, or cyclin A may activate MCC-inhibited Cdc20 even after it has bound to APC/C [92]. The different degradation pathways of cyclins A and B enforced by the SAC likely explain at least a portion of the staggered degradation of the K562 cyclins. However, incorporating these mechanisms in a computational model is difficult at present. Progress has been made in dissecting the full molecular details of the SAC, and several simplified models have been constructed [93], but a more refined description requires additional quantitative data to clarify crucial gaps in pathway knowledge (for example, how SAC inhibition is relieved and the MCC dissociates), and additional modeling approaches may be required to couple the mass action style ODE models explored here with the biophysical modeling of microtubule forces and spatial distributions of molecules, required to account for the fine structural mechanisms underlying the SAC [94].

A much simpler explanation might also be sufficient for explaining the staggered degradations of cyclins A and B. Unlike other cyclins, synthesis of cyclin B has been found to continue through mitosis [95]. The G2 transcription factor, shared between cyclins A and B is currently modeled such that it degrades as cells enter mitosis. Introduction of an additional source of synthesis, here presumed to be dependent on active cyclin B/Cdk1, can effectively prolong cyclin B expression through mitosis and delay its degradation by APC/Cdc20. This is demonstrated in Figure 5. The mechanics underlying this continued synthesis are complicated, but including a synthesis rate proportional to the concentration of active cyclin B/Cdk1 provides a convenient way to model continued expression of cyclin B through mitosis and serves as a placeholder for future, more mechanistically detailed studies.

An additional modification was made to the model that does not affect the simulation output but improves the model’s fidelity with known biology and allows the model to be easily extensible. The Csikasz-Nagy model only considered one population of cyclin A. However, cyclin A is known to bind both Cdk1 and Cdk2. Cyclin A preferentially binds Cdk2, which was shown to be enforced by differential activation by CAK (cyclin H/Cdk7) until a significant fraction of available Cdk2 is bound in complex [96]. Considerable cyclin A binds Cdk1 only as the level of cyclin A/Cdk2 reaches a threshold in late S. However, Cdk2 has been estimated to be present at levels at least 8-times the maximum levels of either cyclin E or cyclin A [97]. Whether the level of cyclin A/Cdk2 truly reaches a plateau, or whether this is an artifact of the measurement process, remains to be determined. Most previous models, including Csikasz-Nagy, did not explicitly consider the binding of cyclins to Cdks, as Cdk levels are relatively constant and in excess of their cyclin partners [97]. Binding and activation is presumed to occur sufficiently fast as to be ignored. Similarly, in our model, we propose and modeled the synthesis of two distinct cyclin A fractions. The binding preference for Cdk2 was enforced by appropriately proportioning the two synthesis rates. Presumably, the two fractions perform separate regulatory tasks. However, little evidence exists as to their distinct functions. We’ve modeled the two fractions as preparation for future efforts, which can further refine the model once more is known about Cyclin A/Cdk2 and Cdk1 specific functions. For now, both are modeled as acting in unison. In keeping with the Csikasz-Nagy model, we did not consider inhibitory phosphorylation of cyclin A/Cdk2, which was shown to be unimportant in unperturbed cells [98]. Similarly, we considered the inhibition cyclin A/Cdk1 by Wee1 and activation by Cdc25 to be insignificant [99]–[101].

By including these mechanisms and tuning several relevant parameters (explained in Materials and Methods), we were able to obtain an improved fit to data, capturing the key data characteristics discussed earlier. These results are presented in Figure 5. Although the fits are significantly improved over earlier models, we think there are parts of the data that illustrate a need for more complexity. The S phase of cyclin A expression in the model is shifted slightly to a later time (Figure 5A); both cyclin A2 and B1 expression data display a rapid initial increase that becomes slightly asymptotic before assuming a linear or exponential S phase rise (Figure 5D, 5E). Finally, while the decay of cyclins A and B in the model occur at the correct time, the exact shape of this decay is not the shape of the data. While there is some variability that is evident in the data, the shape of the decay curve is likely to be accurate [57]. Despite these shortcomings, forcing the model to represent correlated, dense data did drive model modification, and overall, the current model better represents the data.

**Figure 6:**
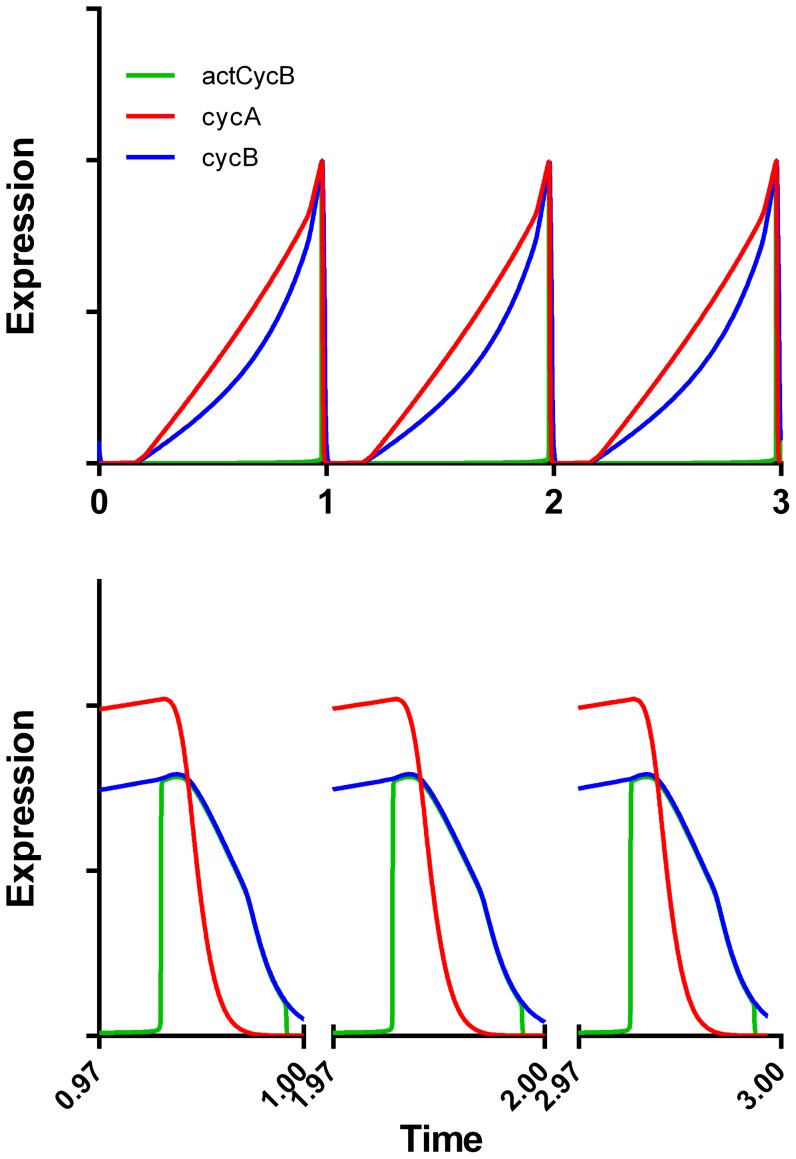
Simulated expression over three successive cell cycles. Model outputs were normalized to zero and one prior to plotting. cycB = total B cyclin; cycA = total A cyclin, and actCycB = active B cyclin (CycB:Cdk1 in Figure 4).

We performed some in silico tests of the model. The first was to determine that the model was stable by setting it up to repeat cycles (Figure 6). This required resetting some G1 variables to starting conditions. These included the delayed response genes (DRG) and D cyclin variables. Significantly, our modifications did not introduce instabilities. Second, we performed D and E cyclin elimination experiments as described by Csikasz-Nagy et al (Figure 8C, 8D, [26]). These experiments lengthened G1, demonstrating that our model maintained the behavior of the Csikasz-Nagy model (Figure S1).

## Discussion

Comparing data and models, we found a mismatch between expression data for two mitotic cyclins from an asynchronously growing human hematopoietic cell line and the state variable output from previously published computational models. Extensive calibration attempts were unable to tune the model parameters and improve the fit. We therefore created a model that combined features of both published models. This was done to cover the important expression features in S, G2, and M phases. Past that, it was necessary to add mechanistic features to improve the correspondence between output and data. We believe this to be a significant forward step, in that it tests the idea that this type of data can drive model synthesis (as opposed to direct measurements of rate constants that are much harder to obtain). Including additional mechanisms improved the model fit to data and provides a starting point for experimental validation of the new ideas introduced by modeling. Mathematical modeling formalizes descriptive knowledge and helps to understand data that represent a large network of interacting variables. Comparing an existing model to data, we discovered previously unmodeled dynamics to be significant factors determining the dynamic expression profiles of cyclins A2 and B1. This emphasizes the importance of data, and indicates the value of data obtained by our methodology [55], [57]. The majority of these model additions are well-supported by published biological experiments. However, the cyclin A-dependent activation of a G2 transcription factor, shared by both cyclins A and B, represents one model imposed hypothesis that needs to be tested. We’ve shown this hypothesis to be consistent with dynamic expression profiles in unperturbed cells, and previously published observations do not contradict it. Modeling therefore also generated a testable hypothesis.

Mathematical approximations represent one level of the hierarchy of modeling assumptions. A simple cyclin A/Cdk-activated transcription factor that abruptly “turns on” at the start of G2 (due to an ultrasensitive, Goldbeter-Koshland switching function) is a simplification of the biochemistry. Such simplifications are limitations of the model. They allow us to reason about the organization of the cell cycle regulatory network and the relationship of our measured markers to that system, but do not generate the highly refined expression data we’ve observed. Future work requires models that are more complex, based on information that is available now, but such models invoke a need for significantly more data for model calibration. The system of data generation is described in two papers [55], [57], one of which provided the data used here. This approach provides correlated data that covers the different time scales and many state variables of the whole system. A current aim would be a better understanding of transcriptional regulation of cyclins A and B, and the reflection of this understanding in working, complex models. An area that needs to be addressed is the use of an exponential increase in E2F activity, which we modeled based on the ability of E2F to activate its own transcription, and which required a commensurate mechanism to reduce E2F activity to a reproducible low level at the end of the cycle, much like the cyclins.

A common method of validating models is to simulate perturbation experiments that have known outcomes. We performed two simulations that eliminate cyclin D or cyclins D and E to confirm that the outputs agree with those of Csikasz-Nagy et al (Figure 8C, 8D, [26]). The outputs from our model are presented in Figure S1 and show that we have not substantively changed the G1 aspects of the Csikasz-Nagy model except as modified by Conradie et al [25]. Thus, we infer that other findings of Csikasz-Nagy remain valid for our model.

Here, our purpose was not to produce a drastically altered, comprehensive cell cycle model. Our purpose was to test the value of the expression data obtained through multiparametric cytometry. We focused on mitotic cyclins because the chances that the underlying model would describe the expression was high. The same is not true for the regulatory system describing early control of the cell cycle. For example, expression of D and E cyclins in cell line models, especially tumor derived lines, is often regulated differently than in primary cells. Real expression data for cyclin E demonstrates that it peaks at the G1/S interface then decreases through S phase to low levels in G2. For many tumor-derived cell lines, cyclin E expression reaches a maximum at G1/S but remains high through most of the cell cycle [102]. The two base models that we used each produce outputs reflecting the unimodal, skewed cyclin E expression of primary cells except that in Csikasz-Nagy et al., the peak occurs at the beginning of the cycle (Figure 8A, 8E, [26]), and in Conradie et al. the peak occurs in G1, just after the restriction point. S begins when half of cyclin E is degraded and degradation is complete by mid-S (Figure 2 in [25]). In our model, cyclin E expression remains very much like the Conradie model except that our S phase expression is higher. Real cyclin E expression in K562 cells appears to peak at the G1/S interface; degrade in early S, then level off at abnormal, high levels [103]. Therefore, all three models get cyclin E expression right in broad concept and wrong in the details. We think that this is the nature of cell cycle modeling without dense, quantitative expression data. Currently, our model could be improved significantly by a data-focused effort to improve the mechanisms that move cells from a pre-committed to committed state in G1 (restriction point). The drive would be to remove the sharp, linked transitions that define Cdh1 loss, cyclin E gain, and improve the onsets of cyclin A2 and cyclin B1 expression in G1.

Currently, quantitative expression data in absolute terms for human cell cycle regulatory proteins are limited. While estimates are available for a small subset of proteins [56], [97], the vast majority are unknown. Frisa and Jacobberger provided a technique to couple absolute concentration calculations with the multi-parametric flow cytometric methods discussed previously [56]. Until such methods are fully implemented, the outputs of the discussed models are in terms of arbitrary units of magnitude, and therefore, the rate constants of the models only capture the time-scales of processes. When quantitative concentration data are available, this information can be incorporated into the model by appropriately scaling the magnitudes of the kinetic rate constants for the reactions in which a given protein participates.

In this model, we assumed a single compartment, freely mixed, aqueous solution (as do all mass action -based models). However, the cell cycle regulatory network (like all events in a cell), is compartmentalized and includes both solution and solid state chemistry. The model does not consider details such as the shuttling of proteins between the nucleus and cytosol or the localization of proteins to solid substrates (such as cyclin B association with the centrosome and mitotic spindle). As it stands, these events are implicit in the equations and rate constants of the vast majority of published models. We’ve sought to improve existing models through dense, quantitative data as a driving force. Future data-driven models could incorporate simplistic compartments (plasma membrane, cytosol, nuclear membrane, nucleus, centrosome) through similar data acquisition by laser scanning cytometry [104].

Finally, single cell measurements of an asynchronous population were used to derive a median expression profile. The corresponding model therefore simulates a “typical” cell of this population. Cytometry data provide some information on the distribution of values in individual cells in a population, and so future work could involve expanding simulation architectures to model the variation of cells about the median trajectory.

Added note: Two recent papers illustrate additional concepts, technology, and algorithms to extract time and expression from static data [105], [106].

## Materials and Methods

Cell line and culture conditions: K562 cells were grown in RPMI 1640 with 10% fetal bovine serum at 5% CO_2_, at 37°C. Cells were kept at or below 10^6^ ml. K562 cells [58], [107] were a gift from Keith Shults and are available from ATCC (www.atcc.org) or DSMZ (www.dsmz.de).

Fixation and Staining: Aliquots of 2×10^6^ cells were fixed with 0.25% formaldehyde followed by 90% MeOH then stored at −20°C. Washed, fixed cells were stained with antibodies to cyclin B1, cyclin A2, and phospho-S10-histone H3 by indirect and direct staining as described [55].

Cytometry and expression profile extraction: Measurements were made with an XL (Beckman Coulter, Miami, FL) or LSR II (BD Biosciences, San Jose, CA). Instrument filters were stock configurations. The expression profiles were extracted as described [55].

Cell cycle analysis on DNA content measurement was performed with ModFit LT 3.0 (Verity Software House, Topsham, ME). Mitotic cell frequency was measured by bivariate analysis of DNA content and PHH3 expression and enumeration of PHH3 positive, 4C cells. WinList 7.0 was used (Verity Software House).

The Systems Biology Toolbox 2 and SBPD packages for MATLAB [108] were used for calibration. Both automated (particularly the simulated annealing, genetic, and SRES algorithms) and manual approaches were used. The modified model presented in this paper was calibrated manually and iteratively as model changes were introduced.

The first model we examined, by Conradie et al. [25], was composed of 23 ordinary differential equations (ODEs) and 74 kinetic rate constants. The mammalian implementation of Csikasz-Nagy et al.’s cell cycle model [26] was composed of 13 ODEs and 66 kinetic rate constants. The Csikasz-Nagy model was modified, combined with the initiating portion of the Conradie model, and expanded to include 25 ODEs and 103 kinetic rate constants. The equations and kinetic rate constant values are provided in Table S3 in File S2. Equations were solved in MATLAB using the ode15s solver.

Data and model output collection and processing were done with Microsoft Excel 2010 (Redman, WA). GraphPad Prism 6.03 (La Jolla, CA) was used for normalization and plotting.

## Supporting Information

Figure S1 **Replication of the experiments performed with the Csikász-Nagy model.** To test whether the structural changes that we introduced in our model have substantively changed G1 behavior, we performed the experiments described in Figure 8C, D, and E of Csikász-Nagy et al. [26]. The “wild type” (WT) cell cycle time for our model was set to 1. **A** shows output for the WT condition (left: entire cycle; right: time period covering “mitosis”). **B** shows cyclin D was “deleted” (ΔD). This corresponds to Figure 8C in [26]. The effect when compared to the unperturbed cycle demonstrates a severe lengthening of the “G1” period that is partially rectified by the contraction of the committed period. **C** shows the results of deleting both cyclins D and E (ΔDΔE). This corresponds to Figure 8D in [26] and results in a similar but more profound first effect (compared to ΔD) that is also partially rectified by contraction of the committed period of the cycle. Both the ΔD and ΔDΔE effects are similar to those of Csikász-Nagy et al. [26]. Color coding and variable names are as in Csikász-Nagy et al. The mapping is CKI = Kip1; actCycE = CycE:Cdk2; Adj CycA = CycA:Cdk1,2; Adj actCycB = CycB:Cdk1, and Cdh1 = Cdh1.
(TIF)

File S1 **Contains the files:** **Table S1.** Classification of published cell cycle models. **Table S2.** Scope of published mammalian cell cycle models.
(PDF)

File S2 **Contains the file: Table S3.** Model Description.
(PDF)
