# Supplementary figures and images for: A Data-Driven, Mathematical Model of Mammalian Cell Cycle Regulation
Source: PLoS One. 2014 May 13;9(5):e97130. doi: 10.1371/journal.pone.0097130 (PMC4019653; doi:10.1371/journal.pone.0097130)

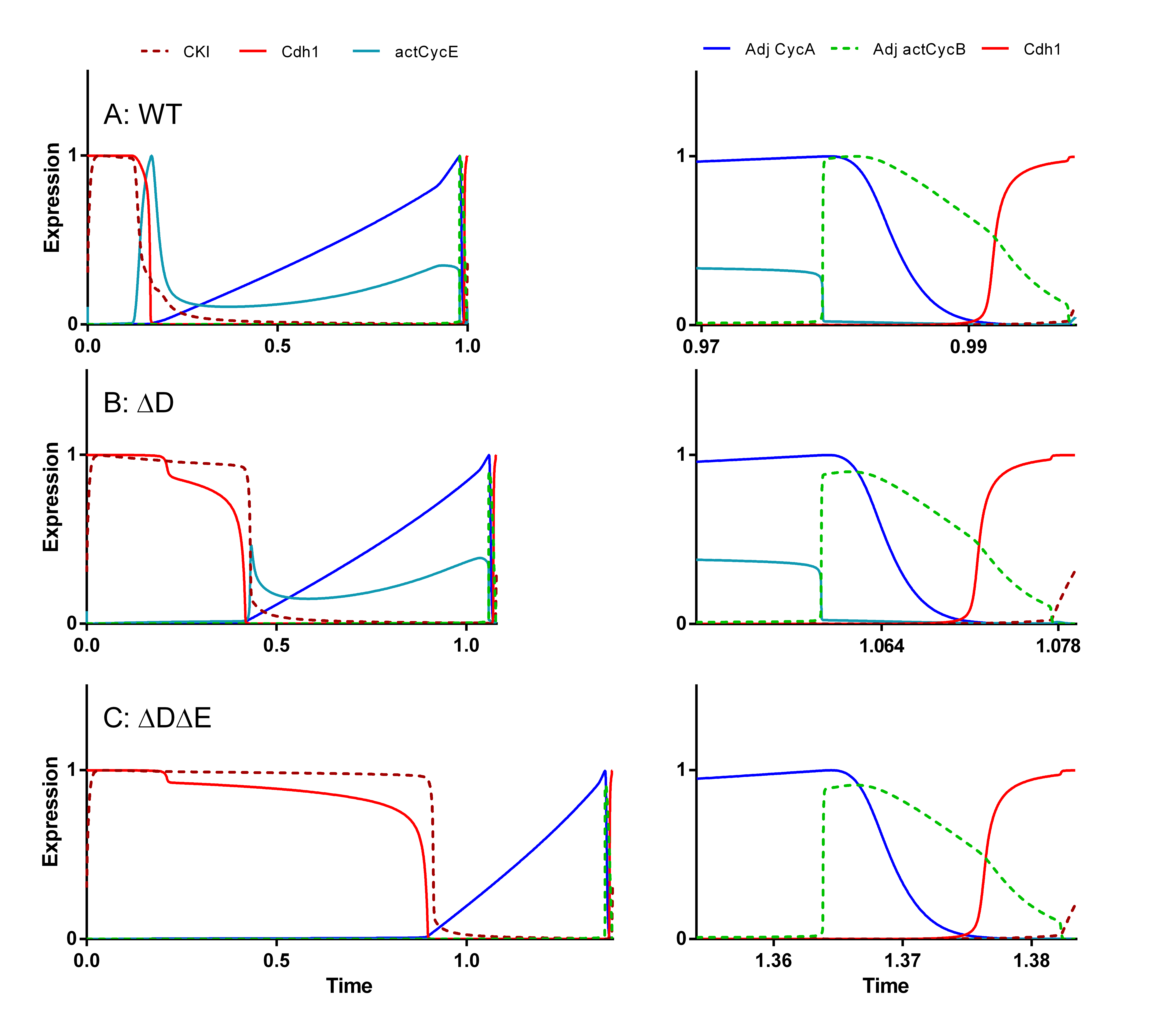

Supplement: Figure S1 — Replication of the experiments performed with the Csikász-Nagy model. To test whether the structural changes that we introduced in our model have substantively changed G1 behavior, we performed the experiments described in Figure 8C, D, and E of Csikász-Nagy et al. [26]. The “wild type” (WT) cell cycle time for our model was set to 1. A shows output for the WT condition (left: entire cycle; right: time period covering “mitosis”). B shows cyclin D was “deleted” (ΔD). This corresponds to Figure 8C in [26]. The effect when compared to the unperturbed cycle demonstrates a severe lengthening of the “G1” period that is partially rectified by the contraction of the committed period. C shows the results of deleting both cyclins D and E (ΔDΔE). This corresponds to Figure 8D in [26] and results in a similar but more profound first effect (compared to ΔD) that is also partially rectified by contraction of the committed period of the cycle. Both the ΔD and ΔDΔE effects are similar to those of Csikász-Nagy et al. [26]. Color coding and variable names are as in Csikász-Nagy et al. The mapping is CKI = Kip1; actCycE = CycE:Cdk2; Adj CycA = CycA:Cdk1,2; Adj actCycB = CycB:Cdk1, and Cdh1 = Cdh1. (TIF) [file pone.0097130.s001.tif]
