# Supplementary material for: A Data-Driven, Mathematical Model of Mammalian Cell Cycle Regulation
Source: PLoS One. 2014 May 13;9(5):e97130. doi: 10.1371/journal.pone.0097130 (PMC4019653; doi:10.1371/journal.pone.0097130)
Supplement: File S1 — Contains the files: Table S1. Classification of published cell cycle models. Table S2. Scope of published mammalian cell cycle models. (PDF) [file pone.0097130.s002.pdf]

Table S1. Classification of published cell cycle models*

Formalism	Prokaryotes or slime mold	Yeast	Xenopus or Drosophila	Mammals	Generic or unspecified
Phenomenological					1-28
Logical/Boolean		29-38		39, 40	
Ordinary differential equation	41-46	32, 43, 47-67	68-84	85-108	42, 99, 108-124
Stochastic		30-33, 50, 55, 56, 61, 65, 67, 125-132		[90, 133, 134]	
Hybrid		61, 135		136	
Delay differential equation		137		138	
Partial differential equation					124

*Citations are repeated for articles containing multiple models of different types.

Table S2. Scope of published mammalian cell cycle models.

Formalism	G/S	G2/M	G1/S/G2/M
Ordinary differential equations	85-93, 95, 97, 100-102, 106	98	94, 96, 103-105, 107, 108
Logical			39, 40
Stochastic	90	134	133
Hybrid			136
Delay differential equations	138		

Note: Since the original compilation of this list, there have been ~28 additional published model papers. These are not relevant to the work presented in this paper. Most seem in line with our manuscript introduction and largely fall within the classification distributions we provided above. Some had models coupled with time course measurements - 2 conducted experiments with yeast and 3 with xenopus extracts.

References

1. Koch AL, Schaechter M (1962) A model for statistics of the cell division process. J Gen Microbiol 29: 435-454.
2. Powell EO (1964) A Note on Koch and Schaechter's Hypothesis About Growth and Fission of Bacteria. J Gen Microbiol 37: 231-249.
3. Smith JA, Martin L (1973) Do cells cycle? Proc Natl Acad Sci U S A 70: 1263-1267.
4. Rossow PW, Riddle VG, Pardee AB (1979) Synthesis of labile, serum-dependent protein in early G1 controls animal cell growth. Proc Natl Acad Sci U S A 76: 4446-4450.
5. Brooks RF, Bennett DC, Smith JA (1980) Mammalian cell cycles need two random transitions. Cell 19: 493-504.
6. Hannsgen KB, Tyson JJ, Watson LT (1985) Steady-state size distributions in probabilistic models of the cell division cycle. SIAM J Appl Math 45: 523-540.
7. Tyson JJ, Hannsgen KB (1985) The distributions of cell size and generation time in a model of the cell cycle incorporating size control and random transitions. J Theor Biol 113: 29-62.
8. Tyson JJ, Diekmann O (1986) Sloppy size control of the cell division cycle. J Theor Biol 118: 405-426.
9. Tyson JJ, Hannsgen KB (1986) Cell growth and division: a deterministic/probabilistic model of the cell cycle. J Math Biol 23: 231-246.
10. Tyson JJ (1989) Effects of asymmetric division on a stochastic model of the cell division cycle. Math Biosci 96: 165-184.
11. Kimmel M, Axelrod DE (1991) Unequal cell division, growth regulation and colony size of mammalian cells: a mathematical model and analysis of experimental data. J Theor Biol 153: 157-180.
12. Basse B, Baguley BC, Marshall ES, Joseph WR, van Brunt B, et al. (2003) A mathematical model for analysis of the cell cycle in cell lines derived from human tumors. J Math Biol 47: 295-312.
13. Gonze D, Goldbeter A (2001) A model for a network of phosphorylation-dephosphorylation cycles displaying the dynamics of dominoes and clocks. J Theor Biol 210: 167-186.

14. Qu Z, Weiss JN, MacLellan WR (2004) Coordination of cell growth and cell division: a mathematical modeling study. J Cell Sci 117: 4199-4207.
15. Castor LN (1980) A G1 rate model accounts for cell-cycle kinetics attributed to 'transition probability'. Nature 287: 857-859.
16. Koch AL (1980) Does the variability of the cell cycle result from one or many chance events? Nature 286: 80-82.
17. Lord PG, Wheals AE (1981) Variability in individual cell cycles of Saccharomyces cerevisiae. J Cell Sci 50: 361-376.
18. Shields R, Smith JA (1977) Cells regulate their proliferation through alterations in transition probability. J Cell Physiol 91: 345-355.
19. Fantes PA, Grant WD, Pritchard RH, Sudbery PE, Wheals AE (1975) The regulation of cell size and the control of mitosis. J Theor Biol 50: 213-244.
20. Fantes PA (1977) Control of cell size and cycle time in Schizosaccharomyces pombe. J Cell Sci 24: 51-67.
21. Shields R, Brooks RF, Riddle PN, Capellaro DF, Delia D (1978) Cell size, cell cycle and transition probability in mouse fibroblasts. Cell 15: 469-474.
22. Sachsenmaier W, Remy U, Plattner-Schobel R (1972) Initiation of synchronous mitosis in Physarum polycephalum. A model of the control of cell division in eukariots. Exp Cell Res 73: 41-48.
23. Smith JA, Martin L (1974) Regulation of cell proliferation. In: Padilla GM, Cameron IL, Zimmerman A, editors. Cell cycle controls: Academic Press. pp. 43-60.
24. Nurse P (1975) Genetic control of cell size at cell division in yeast. Nature 256: 547-551.
25. Johnston GC, Pringle JR, Hartwell LH (1977) Coordination of growth with cell division in the yeast Saccharomyces cerevisiae. Exp Cell Res 105: 79-98.
26. Chiorino G, Metz JA, Tomasoni D, Ubezio P (2001) Desynchronization rate in cell populations: mathematical modeling and experimental data. J Theor Biol 208: 185-199.
27. Nurse P (1990) Universal control mechanism regulating onset of M-phase. Nature 344: 503-508.
28. Lasota A, Mackey MC (1984) Globally asymptotic properties of proliferating cell populations. J Math Biol 19: 43-62.
29. Li F, Long T, Lu Y, Ouyang Q, Tang C (2004) The yeast cell-cycle network is robustly designed. Proc Natl Acad Sci U S A 101: 4781-4786.
30. Stoll G, Rougemont J, Naef F (2006) Few crucial links assure checkpoint efficiency in the yeast cell-cycle network. Bioinformatics 22: 2539-2546.
31. Braunewell S, Bornholdt S (2007) Superstability of the yeast cell-cycle dynamics: ensuring causality in the presence of biochemical stochasticity. J Theor Biol 245: 638-643.
32. Okabe Y, Sasai M (2007) Stable stochastic dynamics in yeast cell cycle. Biophys J 93: 3451-3459.
33. Han B, Wang J (2007) Quantifying robustness and dissipation cost of yeast cell cycle network: the funneled energy landscape perspectives. Biophys J 92: 3755-3763.
34. Faure A, Naldi A, Lopez F, Chaouiya C, Ciliberto A, et al. (2009) Modular logical modelling of the budding yeast cell cycle. Mol Biosyst 5: 1787-1796.
35. Davidich MI, Bornholdt S (2008) Boolean network model predicts cell cycle sequence of fission yeast. PLoS One 3: e1672.
36. Davidich M, Bornholdt S (2008) The transition from differential equations to Boolean networks: a case study in simplifying a regulatory network model. J Theor Biol 255: 269-277.
37. Irons DJ (2009) Logical analysis of the budding yeast cell cycle. J Theor Biol 257: 543-559.
38. Mangla K, Dill DL, Horowitz MA (2010) Timing robustness in the budding and fission yeast cell cycles. PLoS One 5: e8906.
39. Faure A, Naldi A, Chaouiya C, Thieffry D (2006) Dynamical analysis of a generic Boolean model for the control of the mammalian cell cycle. Bioinformatics 22: e124-131.
40. Huang S, Ingber DE (2000) Shape-dependent control of cell growth, differentiation, and apoptosis: switching between attractors in cell regulatory networks. Exp Cell Res 261: 91-103.

41. Tyson J, Kauffman S (1975) Control of mitosis by a continuous biochemical oscillation: synchronization, spatiall inhomogeneous oscillations. J Math Biol 1: 289-310.
42. Tyson JJ (1983) Unstable activator models for size control of the cell cycle. J Theor Biol 104: 617-631.
43. Brazhnik P, Tyson JJ (2006) Cell cycle control in bacteria and yeast: a case of convergent evolution? Cell Cycle 5: 522-529.
44. Li S, Brazhnik P, Sobral B, Tyson JJ (2008) A quantitative study of the division cycle of Caulobacter crescentus stalked cells. PLoS Comput Biol 4: e9.
45. Shen X, Collier J, Dill D, Shapiro L, Horowitz M, et al. (2008) Architecture and inherent robustness of a bacterial cell-cycle control system. Proc Natl Acad Sci U S A 105: 11340-11345.
46. Li S, Brazhnik P, Sobral B, Tyson JJ (2009) Temporal controls of the asymmetric cell division cycle in Caulobacter crescentus. PLoS Comput Biol 5: e1000463.
47. Novak B, Toth A, Csikasz-Nagy A, Gyorffy B, Tyson JJ, et al. (1999) Finishing the cell cycle. J Theor Biol 199: 223-233.
48. Chen KC, Csikasz-Nagy A, Gyorffy B, Val J, Novak B, et al. (2000) Kinetic analysis of a molecular model of the budding yeast cell cycle. Mol Biol Cell 11: 369-391.
49. Ciliberto A, Novak B, Tyson JJ (2003) Mathematical model of the morphogenesis checkpoint in budding yeast. J Cell Biol 163: 1243-1254.
50. Battogtokh D, Tyson JJ (2004) Bifurcation analysis of a model of the budding yeast cell cycle. Chaos 14: 653-661.
51. Chen KC, Calzone L, Csikasz-Nagy A, Cross FR, Novak B, et al. (2004) Integrative analysis of cell cycle control in budding yeast. Mol Biol Cell 15: 3841-3862.
52. Novak B, Tyson JJ (1995) Quantitative analysis of a molecular model of mitotic control in fission yeast. J Theor Biol 173: 283-305.
53. Novak B, Tyson JJ (1997) Modeling the control of DNA replication in fission yeast. Proc Natl Acad Sci U S A 94: 9147-9152.
54. Novak B, Csikasz-Nagy A, Gyorffy B, Chen K, Tyson JJ (1998) Mathematical model of the fission yeast cell cycle with checkpoint controls at the G1/S, G2/M and metaphase/anaphase transitions. Biophys Chem 72: 185-200.
55. Sveiczer A, Csikasz-Nagy A, Gyorffy B, Tyson JJ, Novak B (2000) Modeling the fission yeast cell cycle: quantized cycle times in wee1- cdc25Delta mutant cells. Proc Natl Acad Sci U S A 97: 7865-7870.
56. Sveiczer A, Tyson JJ, Novak B (2001) A stochastic, molecular model of the fission yeast cell cycle: role of the nucleocytoplasmic ratio in cycle time regulation. Biophys Chem 92: 1-15.
57. Novak B, Pataki Z, Ciliberto A, Tyson JJ (2001) Mathematical model of the cell division cycle of fission yeast. Chaos 11: 277-286.
58. Cross FR, Archambault V, Miller M, Klovstad M (2002) Testing a mathematical model of the yeast cell cycle. Mol Biol Cell 13: 52-70.
59. Cross FR (2003) Two redundant oscillatory mechanisms in the yeast cell cycle. Dev Cell 4: 741-752.
60. Thornton BR, Chen KC, Cross FR, Tyson JJ, Toczyski DP (2004) Cycling without the cyclosome: modeling a yeast strain lacking the APC. Cell Cycle 3: 629-633.
61. Barberis M, Klipp E, Vanoni M, Alberghina L (2007) Cell size at S phase initiation: an emergent property of the G1/S network. PLoS Comput Biol 3: e64.
62. Csikasz-Nagy A, Kapuy O, Gyorffy B, Tyson JJ, Novak B (2007) Modeling the septation initiation network (SIN) in fission yeast cells. Curr Genet 51: 245-255.
63. Stelling J, Gilles ED. Robustness vs. identifiability of regulatory modules? the case of mitotic control in budding yeast cell cycle regulation.; 2001 November 2001; Pasadena, CA, USA.
64. Sriram K, Bernot G, Kepes F (2007) A minimal mathematical model combining several regulatory cycles from the budding yeast cell cycle. IET Syst Biol 1: 326-341.
65. Charvin G, Oikonomou C, Siggia ED, Cross FR (2010) Origin of irreversibility of cell cycle start in budding yeast. PLoS Biol 8: e1000284.
66. Li C, Andrake M, Dunbrack R, Enders GH (2010) A bifunctional regulatory element in human somatic Wee1 mediates cyclin A/Cdk2 binding and Crm1-dependent nuclear export. Mol Cell Biol 30: 116-130.

67. Kapuy O, He E, Lopez-Aviles S, Uhlmann F, Tyson JJ, et al. (2009) System-level feedbacks control cell cycle progression. FEBS Lett 583: 3992-3998.
68. Hyver C, Le Guyader H (1990) MPF and cyclin: modelling of the cell cycle minimum oscillator. Biosystems 24: 85-90.
69. Goldbeter A (1993) Modeling the mitotic oscillator driving the cell division cycle. Comments on Theoretical Biology 3: 75-107.
70. Obeyesekere MN, Tucker SL, Zimmerman SO (1992) Mathematical models for the cellular concentrations of cyclin and MPF. Biochem Biophys Res Commun 184: 782-789.
71. Novak B, Tyson JJ (1993) Numerical analysis of a comprehensive model of M-phase control in Xenopus oocyte extracts and intact embryos. J Cell Sci 106 (Pt 4): 1153-1168.
72. Marlovits G, Tyson CJ, Novak B, Tyson JJ (1998) Modeling M-phase control in Xenopus oocyte extracts: the surveillance mechanism for unreplicated DNA. Biophys Chem 72: 169-184.
73. Ciliberto A, Tyson JJ (2000) Mathematical model for early development of the sea urchin embryo. Bull Math Biol 62: 37-59.
74. Ciliberto A, Petrus MJ, Tyson JJ, Sible JC (2003) A kinetic model of the cyclin E/Cdk2 developmental timer in Xenopus laevis embryos. Biophys Chem 104: 573-589.
75. Ciliberto A, Lukacs A, Toth A, Tyson JJ, Novak B (2005) Rewiring the exit from mitosis. Cell Cycle 4: 1107-1112.
76. Pomerening JR, Kim SY, Ferrell JE, Jr. (2005) Systems-level dissection of the cell-cycle oscillator: bypassing positive feedback produces damped oscillations. Cell 122: 565-578.
77. Zwolak JW, Tyson JJ, Watson LT (2005) Globally optimised parameters for a model of mitotic control in frog egg extracts. Syst Biol (Stevenage) 152: 81-92.
78. Calzone L, Thieffry D, Tyson JJ, Novak B (2007) Dynamical modeling of syncytial mitotic cycles in Drosophila embryos. Mol Syst Biol 3: 131.
79. Zwolak J, Adjerid N, Bagci EZ, Tyson JJ, Sible JC (2009) A quantitative model of the effect of unreplicated DNA on cell cycle progression in frog egg extracts. J Theor Biol 260: 110-120.
80. Busenberg S, Tang B (1994) Mathematical models of the early embryonic cell cycle: the role of MPF activation and cyclin degradation. J Math Biol 32: 573-596.
81. Goldbeter A, Guilmot JM (1996) Arresting the mitotic oscillator and the control of cell proliferation: insights from a cascade model for cdc2 kinase activation. Experientia 52: 212-216.
82. Borisuk MT, Tyson JJ (1998) Bifurcation analysis of a model of mitotic control in frog eggs. J Theor Biol 195: 69-85.
83. Pomerening JR, Sontag ED, Ferrell JE, Jr. (2003) Building a cell cycle oscillator: hysteresis and bistability in the activation of Cdc2. Nat Cell Biol 5: 346-351.
84. Tsai TY, Choi YS, Ma W, Pomerening JR, Tang C, et al. (2008) Robust, tunable biological oscillations from interlinked positive and negative feedback loops. Science 321: 126-129.
85. Hatzimanikatis V, Lee KH, Renner WA, Bailey JE (1995) A mathematical model for the G1/S transition of the mammalian cell cycle. Biotechnol Letters 17: 669-674.
86. Obeyesekere MN, Knudsen ES, Wang JY, Zimmerman SO (1997) A mathematical model of the regulation of the G1 phase of Rb+/+ and Rb-/- mouse embryonic fibroblasts and an osteosarcoma cell line. Cell Prolif 30: 171-194.
87. Kohn KW (1998) Functional capabilities of molecular network components controlling the mammalian G1/S cell cycle phase transition. Oncogene 16: 1065-1075.
88. Hatzimanikatis V, Lee KH, Bailey JE (1999) A mathematical description of regulation of the G1-S transition of the mammalian cell cycle. Biotechnol Bioeng 65: 631-637.
89. Obeyesekere MN, Zimmerman SO, Tecarro ES, Auchmuty G (1999) A model of cell cycle behavior dominated by kinetics of a pathway stimulated by growth factors. Bull Math Biol 61: 917-934.
90. Chiorino G, Lupi M (2002) Variability in the timing of G(1)/S transition. Math Biosci 177-178: 85-101.
91. Bai S, Goodrich D, Thron CD, Tecarro E, Obeyesekere M (2003) Theoretical and experimental evidence for hysteresis in cell proliferation. Cell Cycle 2: 46-52.

92. Qu Z, Weiss JN, MacLellan WR (2003) Regulation of the mammalian cell cycle: a model of the G1-to-S transition. Am J Physiol Cell Physiol 284: C349-364.

93. Deineko V, Kel AE, Kel-Margoulis OV, Wingender E, Ratner VA (2003) Simulation of the dynamics of gene networks regulating the cell cycle in mammalian cells. Russian Journal Genetics 39: 1085-1091.

94. Alarcon T, Byrne HM, Maini PK (2004) A mathematical model of the effects of hypoxia on the cell-cycle of normal and cancer cells. J Theor Biol 229: 395-411.

95. Swat M, Kel A, Herzel H (2004) Bifurcation analysis of the regulatory modules of the mammalian G1/S transition. Bioinformatics 20: 1506-1511.

96. Novak B, Tyson JJ (2004) A model for restriction point control of the mammalian cell cycle. J Theor Biol 230: 563-579.

97. Tashima Y, Hamada H, Okamoto M, Hanai T (2008) Prediction of key factor controlling G1/S phase in the mammalian cell cycle using system analysis. J Biosci Bioeng 106: 368-374.

98. Aguda BD, Tang Y (1999) The kinetic origins of the restriction point in the mammalian cell cycle. Cell Prolif 32: 321-335.

99. Aguda BD (1999) A quantitative analysis of the kinetics of the G(2) DNA damage checkpoint system. Proc Natl Acad Sci U S A 96: 11352-11357.

100. Iwamoto K, Tashima Y, Hamada H, Eguchi Y, Okamoto M (2008) Mathematical modeling and sensitivity analysis of G1/S phase in the cell cycle including the DNA-damage signal transduction pathway. Biosystems 94: 109-117.

101. Obeyesekere MN, Herbert JR, Zimmerman SO (1995) A model of the G1 phase of the cell cycle incorporating cyclin E/cdk2 complex and retinoblastoma protein. Oncogene 11: 1199-1205.

102. Pfeuty B, David-Pfeuty T, Kaneko K (2008) Underlying principles of cell fate determination during G1 phase of the mammalian cell cycle. Cell Cycle 7: 3246-3257.

103. Chassagnole C, Jackson RC, Hussain N, Bashir L, Derow C, et al. (2006) Using a mammalian cell cycle simulation to interpret differential kinase inhibition in anti-tumour pharmaceutical development. Biosystems 83: 91-97.

104. Conradie R, Bruggeman FJ, Ciliberto A, Csikasz-Nagy A, Novak B, et al. (2010) Restriction point control of the mammalian cell cycle via the cyclin E/Cdk2:p27 complex. FEBS J 277: 357-367.

105. Gerard C, Goldbeter A (2009) Temporal self-organization of the cyclin/Cdk network driving the mammalian cell cycle. Proc Natl Acad Sci U S A 106: 21643-21648.

106. Alfieri R, Barberis M, Chiaradonna F, Gaglio D, Milanesi L, et al. (2009) Towards a systems biology approach to mammalian cell cycle: modeling the entrance into S phase of quiescent fibroblasts after serum stimulation. BMC Bioinformatics 10 Suppl 12: S16.

107. Gauthier JH, Pohl PI (2011) A general framework for modeling growth and division of mammalian cells. BMC Syst Biol 5: 3.

108. Csikasz-Nagy A, Battogtokh D, Chen KC, Novak B, Tyson JJ (2006) Analysis of a generic model of eukaryotic cell-cycle regulation. Biophys J 90: 4361-4379.

109. Wheals AE (1982) Size control models of Saccharomyces cerevisiae cell proliferation. Mol Cell Biol 2: 361-368.

110. Norel R, Agur Z (1991) A model for the adjustment of the mitotic clock by cyclin and MPF levels. Science 251: 1076-1078.

111. Tyson JJ (1991) Modeling the cell division cycle: cdc2 and cyclin interactions. Proc Natl Acad Sci U S A 88: 7328-7332.

112. Bertuzzi A, De Meo D, Gandolfi A. An oscillator for the control of entry into s and m phases of the cell cycle.; 1997 February, 1997; Rome.

113. Thron CD (1997) Bistable biochemical switching and the control of the events of the cell cycle. Oncogene 15: 317-325.

114. Qu Z, MacLellan WR, Weiss JN (2003) Dynamics of the cell cycle: checkpoints, sizers, and timers. Biophys J 85: 3600-3611.

115. Obeyesekere MN, Tecarro E, Lozano G (2004) Model predictions of MDM2 mediated cell regulation. Cell Cycle 3: 655-661.

116. Yang L, MacLellan WR, Han Z, Weiss JN, Qu Z (2004) Multisite phosphorylation and network dynamics of cyclin-dependent kinase signaling in the eukaryotic cell cycle. Biophys J 86: 3432-3443.
117. Battogtokh D, Tyson JJ (2006) Periodic forcing of a mathematical model of the eukaryotic cell cycle. Phys Rev E Stat Nonlin Soft Matter Phys 73: 011910.
118. Goldbeter A (1991) A minimal cascade model for the mitotic oscillator involving cyclin and cdc2 kinase. Proc Natl Acad Sci U S A 88: 9107-9111.
119. Novak B, Csikasz-Nagy A, Gyorffy B, Nasmyth K, Tyson JJ (1998) Model scenarios for evolution of the eukaryotic cell cycle. Philos Trans R Soc Lond B Biol Sci 353: 2063-2076.
120. Pfeuty B, Kaneko K (2007) Minimal requirements for robust cell size control in eukaryotic cells. Phys Biol 4: 194-204.
121. Thron CD (1991) Mathematical analysis of a model of the mitotic clock. Science 254: 122-123.
122. Gardner TS, Dolnik M, Collins JJ (1998) A theory for controlling cell cycle dynamics using a reversibly binding inhibitor. Proc Natl Acad Sci U S A 95: 14190-14195.
123. Tyson JJ, Novak B (2001) Regulation of the eukaryotic cell cycle: molecular antagonism, hysteresis, and irreversible transitions. J Theor Biol 210: 249-263.
124. Yang L, Han Z, Robb MacLellan W, Weiss JN, Qu Z (2006) Linking cell division to cell growth in a spatiotemporal model of the cell cycle. J Theor Biol 241: 120-133.
125. Steuer R (2004) Effects of stochasticity in models of the cell cycle: from quantized cycle times to noise-induced oscillations. J Theor Biol 228: 293-301.
126. Mura I, Csikasz-Nagy A (2008) Stochastic Petri Net extension of a yeast cell cycle model. J Theor Biol 254: 850-860.
127. Sabouri-Ghomi M, Ciliberto A, Kar S, Novak B, Tyson JJ (2008) Antagonism and bistability in protein interaction networks. J Theor Biol 250: 209-218.
128. Zhang Y, Qian M, Ouyang Q, Deng M, Li F, et al. (3006) Stochastic model of the yeast cell-cycle network. Physica D: Non-linear Phenomena 219: 35-39.
129. Ge H, Qian H, Qian M (2008) Synchronized dynamics and non-equilibrium steady states in a stochastic yeast cell-cycle network. Math Biosci 211: 132-152.
130. Kar S, Baumann WT, Paul MR, Tyson JJ (2009) Exploring the roles of noise in the eukaryotic cell cycle. Proc Natl Acad Sci U S A 106: 6471-6476.
131. Barik D, Baumann WT, Paul MR, Novak B, Tyson JJ (2010) A model of yeast cell-cycle regulation based on multisite phosphorylation. Mol Syst Biol 6: 405.
132. Barik D, Paul MR, Baumann WT, Cao Y, Tyson JJ (2008) Stochastic simulation of enzyme-catalyzed reactions with disparate timescales. Biophys J 95: 3563-3574.
133. Zamborszky J, Hong CI, Csikasz Nagy A (2007) Computational analysis of mammalian cell division gated by a circadian clock: quantized cell cycles and cell size control. J Biol Rhythms 22: 542-553.
134. Kapuy O, He E, Uhlmann F, Novak B (2009) Mitotic exit in mammalian cells. Mol Syst Biol 5: 324.
135. Barberis M, Klipp E (2007) Insights into the network controlling the G1/S transition in budding yeast. Genome Inform 18: 85-99.
136. Singhania R, Sramkoski RM, Jacobberger JW, Tyson JJ (2011) A hybrid model of mammalian cell cycle regulation. PLoS Comput Biol 7: e1001077.
137. Srividhya J, Gopinathan MS (2006) A simple time delay model for eukaryotic cell cycle. J Theor Biol 241: 617-627.
138. Haberichter T, Madge B, Christopher RA, Yoshioka N, Dhiman A, et al. (2007) A systems biology dynamical model of mammalian G1 cell cycle progression. Mol Syst Biol 3: 84.
