# Supplementary material for: A Data-Driven, Mathematical Model of Mammalian Cell Cycle Regulation
Source: PLoS One. 2014 May 13;9(5):e97130. doi: 10.1371/journal.pone.0097130 (PMC4019653; doi:10.1371/journal.pone.0097130)
Supplement: File S2 — Contains the file: Table S3. Model Description. (PDF) [file pone.0097130.s003.pdf]

Table S3. Model Description

Equations:

d(ERG)/dt	=	$\frac{k15}{1+(DRG/J15)^2} - k16 * ERG$	(1)
d(DRG)/dt	=	$k17p * ERG + \frac{k17*(DRG/J17)^2}{1+(DRG/J17)^2} - K18 * DRG$	(2)
d(ppRB)/dt	=	$v29 + v30 + v43 - v44$	(3)
d(E2F)/dt	=	$v29 + v45 + v47 - v46 - v48 + ke2f * E2F * mass - kde2fcdc20 * E2F * Cdc20A$ $-kde2fcdh1 * E2F * Cdh1$	(4)
d(pE2F)/dt	=	$v30 + v49 + v46 - v47 - v50 - kde2fcdc20 * pE2F * Cdc20A - kde2fcdh1 * pE2F * Cdh1$	(5)
d(Rb)/dt	=	$v44 + v45 + v49 - v48 - v50 - v43$	(6)
d(E2FRB)/dt	=	$v51 + v48 - v52 - v29 - v45$	(7)
d(pE2FRB)/dt	=	$v52 + v50 - v51 - v30 - v49$	(8)
d(actCycD)/dt	=	$k9 * DRG + Vdi * TriD + k24r * TriD - k24 * actCycD * freeCKI - k10 * actCycD$	(9)
d(TriD)/dt	=	$k24 * actCycD * freeCKI - k24r * TriD - Vdi * TriD - k10 * TriD$	(10)
d(actCycACdk1)/dt	=	$a1frac * ((ksap + ksapp * E2F + ksappp * TFAB) * mass * 2 + (Vdi + kdia) * TriA)$ $-(Vda + kasa * freeCKI) * actCycACdk1$	(11)
d(actCycACdk2)/dt	=	$(1 - a1frac) * ((ksap + ksapp * E2F + ksappp * TFAB) * mass * 2 + (Vdi + kdia) * TriA)$ $-(Vda + kasa * freeCKI) * actCycACdk2$	(12)
d(actCycB)/dt	=	$Vsb * mass * 2 + V25 * (cycB - actCycB) - (Vdb + Vwee) * actCycB$	(13)
d(actCycE)/dt	=	$(ksep + ksepp * E2F) * mass * 2 + (Vdi + kdie) * TriE - (Vde + kase * freeCKI) * actCycE$	(14)
d(cycA)/dt	=	$(ksap + ksapp * E2F + ksappp * TFAB) * mass * 2 - Vda * cycA$	(15)
d(cycB)/dt	=	$Vsb * mass * 2 - Vdb * cycB$	(16)
d(cycE)/dt	=	$(ksep + ksepp * E2F) * mass * 2 - Vde * cycE$	(17)
d(CKI)/dt	=	$Vsi - Vdi * CKI$	(18)
d(Cdh1)/dt	=	$\frac{(kah1p+kah1pp*Cdc20A)*(1-Cdh1)}{Jah1+1-Cdh1} - \frac{(kih1pp*(actCycACdk1+actCycACdk2)+kih1ppp*actCycB)*Cdh1}{Jih1+Cdh1}$	(19)
d(preMPF)/dt	=	$Vwee * (cycB - preMPF) - (V25 + Vdb) * preMPF$	(21)
d(TriA)/dt	=	$kasa * (cycA - TriA) * freeCKI - (kdia + Vda + Vdi) * TriA$	(22)
d(APCP)/dt	=	$\frac{kaAPC*actCycB*(1-APCP)}{JaAPC+1-APCP} - \frac{kiAPC*APCP}{JiAPC+APCP}$	(23)
d(Cdc20A)/dt	=	$\frac{ka20*APCP*(Cdc20T-Cdc20A)}{Ja20+Cdc20T-Cdc20A} - (\frac{ki20}{Ji20+Cdc20A} + kd20) * Cdc20A$	(24)
d(Cdc20T)/dt	=	$(ks20pp * actCycB)/(J20 + actCycB) - kd20 * Cdc20T$	(25)
d(mass)/dt	=	$u * mass$	(26)

Definitions:

$v29$	=	$E2FRB * (K20 * ((actCycD + TriD) * LD + LA * (actCycACdk1 + actCycACdk2)$ $+LB * actcycB + LE * actCycE))$
$v30$	=	$pE2FRB * (K20 * (LD * (actCycD + TriD) + LA * (actCycACDk1 + actCycACdk2)$ $+LB * actCycB + LE * actCycE))$
$v43$	=	$RB * (K20 * (LD * (actCycD + TriD) + LA * (actCycACdk1 + actCycACdk2)$ $LB * actCycB + LE * actCycE))$
$v44$	=	$ppRB * (K19a * (PP1T - PP1A) + K19 * PP1A)$
$v45$	=	$K26R * E2FRB$
$v46$	=	$E2F * (K23a * (actCycACdk1 + actCycACdk2) + K23b * actCycB)$
$v47$	=	$K22 * pE2F$
$v48$	=	$K26 * E2F * RB$
$v49$	=	$K26R * pE2FRB$
$v50$	=	$K26 * RB * pE2F$
$v51$	=	$K22 * pE2FRB$
$v52$	=	$E2FRB * (K23a * (actCycACdk1 + actCycACdk2) + K23b * actCycB$
$Vatf$	=	$katfpp * (actCycACdk1 + actCycACdk2) + katfppp * actCycE + katfpppp * actCycD$
Vde	=	$kdep + kdepp * actCycE + kdeppp * (actCycACdk1 + actCycACdk2) + kdepppp * actCycB$
Vda	=	$kdap + kdapp * Cdc20A + kacdh1 * Cdh1$
$TFAB$	=	$G(kafab * (actCycACdk1 + actCycACdk2), kifb, Jafb, Jifb)$
Vsi	=	$ksip$
Vsb	=	$ksbp + ksbpp * TFAB + ksbppp * actCycB + ksbppp * E2F$
Vdb	=	$kdbp + kdbpp * Cdh1 + kdbppp * Cdc20A$
$Wee1$	=	$G(kaweep, kiwee * (actCycACdk1 + actCycACdk2) + kiweeb * actCycB, Jawee, Jiwee)$
$Vwee$	=	$kweep + kweepp * Wee1$
$Cdc25$	=	$G(ka25 * actCycB, ki25p, Ja25, Ji25)$
$V25$	=	$k25p + k25pp * Cdc25$
Vdi	=	$(kdip + kdipp * (actCycACdk1 + actCycACdk2) + kdippp * actCycB + kdipppp * actCycE)$
$TriE$	=	$cycE - actCycE$
$freeCKI$	=	$CKI - TriA - TriE - TriD$
$CdkCycBCKI$	=	$cycB - actCycB - preMPF$
$Cdk1PCycB$	=	$cycB - actCycB$
$PP1A$	=	$PP1T\ /\ K21 * (FE * (actCycACdk1 + actCycACdk2 + actCycE) + FB * actCycB + 1)$

Where G(…) is the Goldbeter-Koshland Function:

$$B(A_1, A_2, A_3, A_4) = A_2 - A_1 + A_3 * A_2 - A_4 * A_1$$

$$G(A_1, A_2, A_3, A_4) = \frac{2 * A_4 * A_1}{B(A_1, A_2, A_3, A_4) + \sqrt{B(A_1, A_2, A_3, A_4) - 4 * (A_2 - A_1) * A_3 * A_1}}$$

Kinetic Rate Constants:

a1frac	=	0.081283	kah1p	=	155.8708	ksep	=	1.562461
FB	=	2	kah1pp	=	176350	ksepp	=	8.8175
FE	=	25	kasa	=	19733.57	ksip	=	390.9926
J15	=	0.1	kase	=	19733.57	kweep	=	234.8312
J17	=	0.3	katfpp	=	58.70692	kweepp	=	17635
J20	=	100	katfppp	=	97.80724	LA	=	30
Ja20	=	0.005	katfpppp	=	77.63932	LB	=	0.5
Ja25	=	0.005	kaweep	=	13.8188	LD	=	3.3
JaAPC	=	0.01	kd20	=	17.635	LE	=	10
Jafb	=	0.01	kdap	=	0.516094	PP1T	=	1
Jah1	=	0.15	kdapp	=	2645.25	u	=	0.693937
Jatf	=	0.01	kdbp	=	0.853181			
Jawee	=	0.05	kdbpp	=	176.35			
Jaweeb	=	0.05	kdbppp	=	387.97			
Ji20	=	0.005	kde2fcdc20	=	881.75			
Ji25	=	0.031623	kde2fcdh1	=	1.7635			
JiAPC	=	0.001	kdep	=	1.961012			
Jifb	=	0.001	kdepp	=	1.973357			
Jih1	=	0.01	kdeppp	=	176.35			
Jitf	=	0.01	kdepppp	=	3527			
Jiwee	=	0.05	kdia	=	196.0783			
k10	=	88.175	kdie	=	196.0783			
k15	=	5.2905	kdip	=	196.0783			
k16	=	44.0875	kdipp	=	978.0688			
k17	=	2645.25	kdippp	=	1960.837			
k17p	=	2.64525	kdipppp	=	978.0688			
k18	=	176.35	ke2f	=	4.2324			
K19	=	35.27	ki20	=	17.635			
K19a	=	440.875	ki25p	=	35.27			
K20	=	176.35	kiAPC	=	3.862259			
K21	=	1	kifb	=	9.827456			
K22	=	3.527	kih1pp	=	17635			
K23a	=	0.17635	kih1ppp	=	1763.5			
K23b	=	1.7635	kitfp	=	48.96181			
k24	=	1763.5	kitfpp	=	19.60836			
k24r	=	176.35	kitfppp	=	19.60836			
k25p	=	61.474	kiwee	=	0.145			
k25pp	=	30515.96	kiweeb	=	5			
K26	=	17635	ks20pp	=	105.81			
K26R	=	35.27	ksap	=	16.75325			
k9	=	45.851	ksapp	=	0.10581			
ka20	=	292.669	ksappp	=	20.28025			
ka25	=	8.85277	ksbp	=	6.7013			
kaAPC	=	2.33401	ksbpp	=	15.8715			
kacdh1	=	264.525	ksbppp	=	1.7635			
kafab	=	0.296268	ksbpppp	=	0.617225			
